# Cervical Cancer Knowledge and Screening Patterns in Zuni Pueblo Women in the Southwest United States

**DOI:** 10.1007/s13187-023-02295-8

**Published:** 2023-04-13

**Authors:** Kate Cartwright, Mikaela Kosich, Madison Gonya, Deborah Kanda, Samantha Leekity, Judith Sheche, Nicholas Edwardson, V. Shane Pankratz, Shiraz I. Mishra

**Affiliations:** 1 School of Public Administration, University of New Mexico, Albuquerque, NM USA; 2 Comprehensive Cancer Center, University of New Mexico, Albuquerque, NM USA; 3 Department of Obstetrics and Gynecology, University of New Mexico, Albuquerque, NM USA; 4 Department of Internal Medicine, University of New Mexico, Albuquerque, NM USA; 5 Departments of Pediatric and Family and Community Medicine, University of New Mexico, Albuquerque, NM USA

**Keywords:** Cervical cancer, Cancer screening, American Indian/Alaska Native, Health equity

## Abstract

American Indian women experience cervical cancer disparities, including later-stage diagnosis and a higher cervical cancer mortality rate. These disparities are interconnected and linked to cervical cancer screening disparities. Cervical cancer when identified early is highly treatable. Individual- and health system-level factors often contribute to gaps in cervical cancer screening. To better understand the source of these inequities experienced by American Indian women, specifically Zuni women, this paper examines how knowledge about cervical cancer and related risk factors is linked to cervical cancer screening for Zuni women using primary data gathered by the Zuni Health Initiative in 2020 and 2021. We find that of the women who completed the survey (*n* = 171), women with greater cervical cancer knowledge are statistically significantly more likely to have received cervical cancer screening. Closer examination of knowledge on the specific risk factors for cervical cancer provides evidence upon which to develop a cervical cancer education intervention.

## Introduction

Advances in cervical cancer science enable the prevention of cervical cancer through HPV vaccines and minimally invasive and highly successful treatment through early detection of cervical cancer [15, 26]. These advances have also led to improvements in cervical cancer incidence and mortality for most US women, although cervical cancer disparities exist across a number of factors [1, 11]. Race is a persistent determinant of cancer outcomes, and in New Mexico (NM) and across the Southwestern US, American Indians (AI) experience a range of inequities [16, 17, 25, 26].

According to 2020 BRFSS data, the percentage of women aged 21–65 years old who have had a Pap test in the last 3 years (no hysterectomy) is as follows: 71.63% of American Indian/Alaska Native (AI/AN) women residing in NM, 71.85% of Non-Hispanic White women residing in NM, and 77.9% of US women of all races [22]. Based on Surveillance, Epidemiology, and End Results (SEER) data from 2015 to 2019, the age-adjusted incidence rate of cervical cancer for AI women in NM was 7.2 per 100,000 compared to 8.0 for non-Hispanic White women in NM (NM Tumor [13]). These same data report a cervical cancer mortality rate of 2.0 per 100,000 for AI women in NM compared to 1.7 per 100,000 for non-Hispanic White women in NM (NM Tumor [13]). While these statistics show a similar rate of cervical cancer screening between AI women and non-Hispanic White women in NM and a lower incidence rate of cervical cancer for AI women compared to non-Hispanic White women in NM, the inequity is captured in the greater cervical cancer mortality rate facing AI women. This inequity is also captured in the 2018 and 2019 National Health Interview Surveys and other reports by the CDC [1].

The experience of AI women in relation to cervical cancer screening is one that is understudied compared to the general population of women in the USA, and there are distinct barriers impacting cancer disparities within AI populations [25]. These include geographic, financial, and health system barriers ranging from substantial geographic distance to limited health care services, limited financial resources to facilitate access to health care (such as resources to travel to health care, ability to take time away from work), and bureaucratic barriers such as being able to make an appointment for specialty medical care [19, 25, 26].

Healthcare can make a difference in cervical cancer disparities. First, cervical cancer may be prevented by stronger uptake of the human papillomavirus (HPV) vaccine. Second, through regular Pap and HPV tests, cervical cancers may be identified at very early stages, where it is both easier and more effectively treated. The US Preventative Services Task Force (USPSTF) recommends that women aged 21–65 receive cervical cancer screening [24]. When women are screened in a timely and regular rhythm, preventive screening for cervical cancer is effective in decreasing both incidence and mortality of cervical cancer [15]. Bridging the AI/AN disparities in cervical cancer prevention, including the HPV vaccine, screening, and care, requires stronger evidence and understanding surrounding their experiences [2, 26].

Women and their experiences with cervical cancer screening, preventive medicine, and cancer treatment are culturally and contextually nuanced. Social and cultural norms may also be barriers to timely screening and treatment for cervical cancer. As cervical cancer is linked to sexual and reproductive health, stigma may be a barrier to promoting cervical cancer prevention, screening, and treatment. Specifically, literature shows that the linkage between cervical cancer and HPV leads to heightened stigmatization of women with cervical cancer [14, 20]. In qualitative research with AI/AN communities about cervical and breast cancer, women have reported that stigma can be a barrier to getting care and that screening itself requires people to make themselves vulnerable [9, 21]. Literature also suggests that beliefs around cancer and death can influence people’s willingness to participate in cancer screening [4, 9].

While most population science examines AI/AN health disparities by grouping all AI/AN Tribes together, the AI/AN population is not a monolith and Tribal differences need to be acknowledged and accounted for in cancer prevention plans [11, 16–18, 25]. In this study, we contribute to the evidence base of cervical cancer screening knowledge among the Zuni people, who may have distinctly different, culturally derived knowledge of cervical cancer, which may be different from other AI Tribes. This study aims to examine the association between general cervical cancer awareness, cervical cancer risk factor knowledge, and cervical cancer screening patterns in Zuni Pueblo women.

## Methods

### Research Setting

This project analyzes data from a community-engaged cancer control survey with a priority focus on cervical, breast, and colorectal cancers in Zuni Pueblo, the largest Pueblo Tribe in New Mexico [3, 7]. There are approximately 11,000 Zuni, and the most recent American Community Survey 5-year estimate (2017–2021) of full-time residents in Zuni Pueblo is approximately 7000 [8, 23]. While Zuni is a small, rural community, some preventative cervical cancer care, including Pap/HPV tests, is available locally at the Indian Health Service (IHS) administered Zuni Comprehensive Health Center [8, 23].

### Sampling Strategy and Eligibility Criteria

The sampling strategy for the survey started with a complete enumeration of streets in Zuni, all of which were selected in a random order for recruitment through flyers. This recruitment strategy was supplemented through outreach at high-traffic community locations and public service announcements on the community radio station. Snowball sampling from these contacts and key community stakeholders was also employed, but COVID-19 restrictions limited most in-person recruitment strategies. Eligibility included self-identifying as AI, a member of the Zuni tribe, or married to a Zuni tribal member, and meeting the age and gender requirements for the age/gender-specific survey. This project has received research approval from the Zuni Pueblo Tribal Council, the Southwest Tribal Institutional Review Board (IRB), and UNM Health Sciences Center IRB.

### Study Implementation

The survey used an observational, cross-sectional design. The Zuni Health Initiative (ZHI) staff conducted surveys among eligible adult men ages 50–75 and women ages 21–75, between October 2020 through April 2021, in the Zuni Pueblo. Men ages 50–75 comprise approximately 12% and women ages 21–75 comprise approximately 35% of current Zuni Pueblo residents [23]. The survey varied in length based on the age/gender-specific survey. ZHI staff recruited 281 participants: 61 surveys were completed by women ages 21–49, 110 by women ages 50–75, and 110 by men ages 50–75. All participants received a merchandise card in recognition of their participation in the study. This analysis focuses on the 171 surveys who answered questions about cervical cancer (which includes women ages 21–49 and women ages 50–75). Due to COVID-19 pandemic precautions, surveys were conducted by phone, and any in-person outreach used standard COVID-19 mitigation protocols to protect community health.

### Measures

#### Cervical Cancer Screening

The dependent variable for this study is self-report of ever having had a cervical cancer screening (specifically a Pap or HPV test). Participants were asked if they have ever been screened for cervical cancer with a Pap or HPV test. If they answered yes, further questions were asked about the specific type of test and when they had their most recent test. Due to sample size and the disruption of preventive services due to COVID-19, this analysis focuses on ever being screened for cervical cancer.

#### Cervical Cancer Awareness and Risk Factor Knowledge

The measures include awareness regarding cervical cancer screening and prevention awareness (including the HPV vaccine) and knowledge of cervical cancer risk factors. Items measuring cervical cancer screening and prevention awareness include having talked with a doctor about testing, heard of HPV, and heard of HPV vaccine. Cervical cancer risk factor knowledge is measured by the sum of correct answers to 18 questions focused on knowledge of specific cervical cancer risk factors (see Table 1 for the individual items). Our measures are modeled after prior work on cancer screening awareness and knowledge [16, 17].

**Table 1:** Descriptive statistics (*n*=171)

			Cervical cancer screening		
Characteristic		All	No	Yes	*p*-value*
Age		51.5 (14.15)	43.24 (16.07)	54.19 (12.39)	0.0002
Live-in partner	Yes	85 (49.71%)	20 (47.62%)	65 (50.39%)	0.755
	No	86 (50.29%)	22 (52.38%)	64 (49.61%)	
Education	Less than HS	29 (16.96%)	9 (21.43%)	20 (15.50%)	0.056
	HS	55 (32.16%)	17 (40.48%)	38 (29.46%)	
	More than HS	87 (50.88%)	16 (38.10%)	71 (55.04%) i	
Income	Less than 10k	68 (39.77%)	21 (50%)	47 (36.43%)	0.192
	10k–20k	51 (29.82%)	12 (28.57%)	39 (30.23%)	
	20k+	49 (28.65%)	8 (19.05%)	41 (31.78%)	
Employed	Yes	83 (48.54%)	19 (45.24%)	64 (49.61%)	0.622
	No	88 (51.46%)	23 (54.76%)	65 (50.39%)	
Has regular healthcare provider	Yes	108 (63.91%	19 (53.66%)	90 (69.53%)	0.007
	No	61 (36.09%)	22 (53.66%)	39 (30.47%)	
Cervical cancer screening					
Screening ever	Yes	129 (75.44%)	42 (100%)	129 (100%)	
Screening current	Yes	72 (42.11%)	--	72 (55.81%)	
Last test (of those screened)	Pap test	104 (60.82%)	--	104 (80.62%)	
	HPV test	1 (0.58%)	--	1 (0.78%)	
	Both	24 (14.04%)	--	24 (18.6%)	
General cervical cancer awareness					
Talked to doctor about testing		71 (41.52%)	9 (21.43%)	62 (48.06%)	0.002
Heard of HPV		94 (54.97%)	19 (45.24%)	75 (58.14%)	0.144
Heard of HPV vaccine		56 (32.75%)	13 (30.95%)	43 (33.33%)	0.752
Cervical cancer risk factor knowledge					
Which of these increases the risk of cervical cancer?					
*Medically more reasonable risk factors (Yes=1, No=0)*					
Having relatives with cervical cancer (that is, genetics)		132 (77.19%)	33 (78.57%)	99 (76.74%)	0.8064
Having multiple sex partners		129 (75.44%)	25 (59.52%)	104 (80.62%)	0.0058
Have given birth to three or more children		56 (32.75%)	15 (35.71%)	41 (31.78%)	0.6373
Using birth control pills for a long time (≥5 years)		89 (52.05%)	20 (47.62%)	69 (53.49%)	0.5084
Being sexually active at an early age		112 (65.5%)	26 (61.9%)	86 (66.67%)	0.5729
Smoking or being exposed to cigarette smoke		91 (53.22%)	15 (35.71%)	76 (58.91%)	0.0089
Being < 17 at first full-term pregnancy		52 (30.41%)	9 (21.43%)	43 (33.33%)	0.1452
Having a weakened immune system (e.g., HIV)		130 (76.02%)	29 (69.05%)	101 (78.29%)	0.2228
Being overweight		68 (39.77%)	16 (38.1%)	52 (40.31%)	0.7989
A diet low in fruits and vegetables		85 (49.71%)	24 (57.14%)	61 (47.29%)	0.2672
Knowing HPV causes cervical cancer		117 (68.42%)	24 (57.14%)	93 (72.09%)	0.0636
*Medically less reasonable risk factors (Yes=0, No=1)*					
A diet low in fiber and high in fat		101 (59.06%)	22 (52.38%)	79 (61.24%)	0.3105
A diet high in processed meats		105 (61.4%)	21 (50%)	84 (65.12%)	0.0805
Lack of cleanliness/hygiene		112 (65.5%)	31 (73.81%)	81 (62.79%)	0.192
Vaginal infections		144 (84.21%)	36 (85.71%)	108 (83.72%)	0.7583
Having sexual intercourse during menstruation		70 (40.94%)	17 (40.48%)	53 (41.09%)	0.9444
Receiving hits or bruises in the vagina area		79 (46.2%)	23 (54.76%)	56 (43.41%)	0.2
Using preservatives in food		52 (30.41%)	12 (28.57%)	40 (31.01%)	0.7656
Cervical cancer risk factor knowledge total score	Max score=18	9.33 (1.8)	8.62 (1.99)	9.57 (1.68)	0.0072

*Control variables* include age, income, education, relationship status, and if the participant has a regular healthcare provider.

### Data Analysis

This study begins with descriptive statistics to understand initial patterns between variables and cervical cancer screening. Bivariate associations are tested using the appropriate test (*t*-test or chi-squared). Spearman correlation coefficients estimate the magnitude of these associations. Finally, this study uses logistic regression to identify the variables associated with cervical cancer screening while controlling for key demographic characteristics.

## Results

### Descriptive Characteristics

Table 1 presents the descriptive characteristics of the 171 participants, from the descriptive statistics for the demographic measures to the key dependent and independent variables. Detailed information on individual cervical cancer knowledge responses is also provided.

### Demographics

Respondents range in age from 21 to 75 with a mean age of 51.5 years. Half of the respondents live with a partner and half of the respondents are employed. Approximately 40% report incomes of less than $10,000 annually, approximately 30% report annual incomes between $10,000 and $20,000, and approximately 29% report annual incomes above $20,000. Over 32% of respondents have at least a high school level of education, and 51% of the participants have attained more than a high school level of education. Finally, approximately 64% of respondents report having a regular healthcare provider (Table 1).

### Cervical Cancer Screening

Of the 171 respondents, 75% report having ever been screened for cervical cancer and 42% report being current with cervical cancer screening guidelines. Of those screened, approximately 81% report having a Pap test only, less than 1% report having an HPV test only, and approximately 18% report having both Pap and HPV test (Table 1).

### Cervical Cancer Awareness and Risk Factor Knowledge

Of the participants, approximately 42% report having talked to their doctor about cervical cancer testing, 55% have heard of HPV, and 32% have heard of the HPV vaccine. The mean score of the sum of correct cervical cancer knowledge items is 9.33 (SD 1.8).

### Bivariate Correlations

Table 2 shows correlations between all pairs of variables. Having ever received a cervical cancer screening test is significantly correlated with age, having a regular healthcare provider, having talked to a provider about cervical cancer, and having a higher risk factor knowledge score. Having a higher cervical cancer knowledge score is not significantly correlated with any other measure. While age is positively and significantly correlated with having received a cervical cancer test, it is negatively and significantly correlated with having heard of HPV and having heard of the HPV vaccine. Older women are less likely to have heard of HPV and the HPV vaccine. Having a regular health care provider is positively and significantly correlated not only with receiving a cervical cancer test, but also with age, income, and having spoken to a provider about cervical cancer.

**Table 2:** Correlations among variables (*n*=171)

	1	2	3	4	5	6	7	8	9	10
1. Receipt of cervical cancer screening										
2. Age	0.291***									
3. Live-in partner	−0.024	0.135								
4. Education level	0.146	0.050	−0.064							
5. Household income	0.140	0.056	−0.220**	0.317***						
6. Employment	−0.038	0.210**	0.088	−0.041	−0.121					
7. Regular healthcare provider	0.207**	0.282***	0.106	0.158*	0.183*	0.100				
8. Ever talked to provider about cervical cancer	0.236**	0.186*	−0.022	0.159*	0.188*	−0.032	0.176*			
9. Heard of HPV	0.112	−0.227**	−0.053	0.301***	0.050	−0.056	0.014	0.258***		
10. Heard of HPV vaccine	0.024	−0.250***	−0.050	0.192*	0.167*	0.109	−0.001	0.038	0.404***	
11. Risk factor knowledge	0.190*	0.051	−0.066	−0.025	0.009	0.009	0.022	0.141	0.031	−0.084

*p < 0.05, *p<0.01, ***p<0.001

### Multivariable Predictors of Cervical Cancer Screening

Table 3 shows the results of the logistic regression analysis. Only age and cervical cancer knowledge are statistically significant in the multivariable model: when adjusting for other factors, being 1 year older is associated with 1.07-fold higher odds of reporting having had a screening (95% CI: 1.03–1.10) and having one point higher of a cervical cancer knowledge score is associated with 1.36-fold higher odds of reporting ever having had a screening (95% CI: 1.07–1.73).

**Table 3:** Logistic regression model of ever receiving cervical cancer screening (*n*=164)

	Screening ever	
	Beta (SE)	OR (95%)
Sociodemographics		
Age	0.062 (0.017)***	1.064 (1.028–1.101)
Live-in partner (vs no)	0.052 (0.221)	1.110 (0.466–2.644)
Education beyond HS (vs HS or less)	0.038 (0.241)	1.078 (0.419–2.775)
Income 10k–20k (vs <10k)	−0.026 (0.324)	1.253 (0.452–3.471)
Income >20k (vs <10k)	0.278 (0.368)	1.698 (0.522–5.525)
Employed (vs no)	0.281 (0.222)	1.753 (0.735–4.183)
Regular provider (vs no)	0.221 (0.223)	1.557 (0.649–3.732)
Cervical cancer awareness		
Talked to doctor about testing (vs no)	0.201 (0.246)	1.495 (0.571–3.913)
Heard of HPV (vs no)	0.450 (0.260)	2.458 (0.886–6.814)
Heard of HPV vaccine (vs no)	0.154 (0.265)	1.361 (0.482–3.846)
Cervical cancer risk factor knowledge	0.310 (0.121)*	1.363 (1.075–1.728)
Model chi-square (df=11)	27.598	

*p < 0.05, *p<0.01, ***p<0.001

## Discussion

This study provides important insights into cervical cancer screening knowledge and patterns in Zuni women. Over 75% of Zuni women reported having had at least one cervical cancer screening in their lifetime. This is a strong base to build from, as the literature suggests that awareness of cervical cancer and related screenings are linked to receiving future screenings [14]. A smaller percentage, approximately 55% of those who have ever received a screening (42% of all respondents in this survey), are up to date with cervical cancer screening guidelines. These patterns help provide information about where to target interventions. There should be specific outreach to the 25% of women who have never had a cervical cancer screening to explain the importance of the screening to identify cervical cancer at the earliest possible stage when it is most effectively and least invasively treatable. There should be a different approach for those women who have had a screening in the past but are not currently up to date.

This study was conducted during the COVID-19 pandemic, when there was a downturn in “elective” healthcare and cancer screening more broadly [5, 6]. For example, Zuni Comprehensive Health Center did not provide many primary care services during some of the most critical stages of the pandemic to protect public health. This had an impact on preventive care services. Again, our survey was conducted between October 2020 and April 2021, and during this time over one-third of our participants reported not having a regular provider. However, as healthcare facilities move forward at this stage of the pandemic to operate with fewer restrictions and closer to pre-pandemic routines, it will be important to note if the number of people without regular providers decreases as well.

This study provides further evidence that cervical cancer knowledge is linked to cervical cancer screening behaviors. For these Zuni women, having more knowledge about which factors increase the odds of cervical cancer is linked to having been screened for cervical cancer. Cervical cancer education intervention studies indicate that interventions which increase cervical cancer and screening knowledge improve screening behaviors [12, 14]. Interventions for and evaluations of cancer screening should be culturally tailored. Considering this evidence and our findings about cervical cancer knowledge held by Zuni women, cervical cancer education outreach may result in improving cervical cancer screening uptake for Zuni women. Taking a closer look at the individual cervical cancer knowledge items suggests some areas focus on in education interventions. While an education program should be holistic, there are some areas where there was greater room for knowledge, specifically medically reasonable risk factors. For example, while over 77% of respondents knew that if someone in their family had cervical cancer, it increased their odds of cervical cancer, a smaller percentage knew that if someone had given birth to 3 or more children (33%) and if someone had their first pregnancy younger than 17 years old (30%), it increased their odds of cervical cancer. These responses provide knowledge to build a community-tailored education intervention with the greatest potential for improving cervical cancer knowledge and screening for Zuni women.

While many of the demographic characteristics were not strongly linked to cervical cancer screening, it was not surprising that as women age, they are more likely to receive a screening. It is also noteworthy that younger women are statistically significantly more likely to have heard of HPV and the HPV vaccine, as the medical community has made major advancements in HPV knowledge and prevention in the last 20 years. While there is room for improvement, information about HPV and the HPV vaccine is reaching younger AI/AN women [10]. Hopefully, this pattern will result in promoting HPV and cervical cancer screening in these younger women. Our findings suggest that women of different generations will benefit from tailored education interventions which consider age-specific cultural norms related to reproductive health. This education should focus on HPV education for at-risk populations including education about HPV types, which types are covered by the current vaccine, and to discuss current CDC recommendations [24].

The limitations of this study to consider include that the survey is cross-sectional, so specific causal claims cannot be made, and the sampling strategy was not a strict random sample, limiting the generalizability. A true strength of this study is its focus on Zuni women. While this study makes a contribution to a greater understanding of AI/AN cervical cancer patterns, AI/AN health scholars should honor the unique culture and context of Tribes and apply the takeaways from this study in consideration of specific Tribes’ and communities’ distinct cultures and needs. Finally, this study was conducted during the COVID-19 pandemic, so while it captures distinct patterns about cervical cancer screening, the relatively lower rates of cervical cancer screening compliance may have been affected by the reduction in access to care due to pandemic precautions in the healthcare system and by individuals.

## Conclusion

This study builds the body of evidence about cervical cancer knowledge and screening in Zuni Pueblo. It offers concrete areas upon which to build interventions to improve cervical screening compliance. It reveals both the strong foundations of knowledge within Zuni and provides areas where education efforts may make the most impact. There is great potential to improve cervical cancer knowledge and screening which will reduce the burden of cervical cancer in Zuni women.
